# Supplementary material for: Health system responsiveness and its associated factors for delivery care in public health facilities of West Arsi Zone, Oromia, Ethiopia
Source: PLoS One. 2026 Jan 7;21(1):e0340691. doi: 10.1371/journal.pone.0340691 (PMC12779144; doi:10.1371/journal.pone.0340691)
Supplement: S2 File — (DOCX) [file pone.0340691.s002.docx]

**Annex I: Consent to participate in study:**

Hello! I am --------------------------------- & data collector for the study conducted on “Health System Responsiveness and its associated factors for Delivery Care in Public Health Facilities of West Arsi Zone, Oromia, Ethiopia” which is conducted by investigators from Madda Walabu University, Shashemene Campus. The aim of the study is to assess the health system responsiveness and its associated factors for delivery care in public health facilities of West Arsi Zone, Oromia. If you agree to participate in this study, you will be required to answer a series of question that have been prepared for the study through interview in order to obtain the intended information. The interview will take approximately 25-30 minutes. All information that will be collected will be kept in private and will be used only for this study. The form will not bear your name but identification number. Participating in this study is completely voluntary. You have a right not to participate in this study and even if you have already accept to participated in the study you can quit at any time if you feel so. Refusal to participate or withdrawal from the study will not involve penalty or loss of any benefits.

**Informed Consent Form**

Dear my participant, I would be grateful if you would participate in this study by completing this questionnaire. It hoped that the findings of this study would help to identify factors associated to the health system responsiveness for delivery care in public health facilities of West Arsi Zone, Oromia. Be assured that the information you provide will be used for research purposes only and will be treated as confidential and participation will be based on voluntarily will**.** I also would like to assure you that any time you feel uncomfortable participating on the study and withdrawing from the study. The study does not have any effect on the service you obtain from the respective health facility.

If you would like to know more, please contact:

**Address of the Principal Investigators Name**Negeso Gebeyehu, 0937335386

I thank you in advance for taking your time to answer questions.

Do you agree to participate in this study?

Yes, continue Sign________ Date______ No, thank you!

Name of the data collector_____________________________ Sign________ Date______

Date_________________Start time_____________End time__________________

# **Annex II: Data Collection Tool**

| **Part I –Socio- demographic characteristics** | | | |
| --- | --- | --- | --- |
| **No** | **Questions** | **Responses** | **Skip to** |
|  | Code/MRN | _________________ |  |
|  | How old are you? Age at interview in completed year | _________________ years |  |
|  | What is your current marital status? | 1. Single 2. Married 3. Other(specify)_____________ |  |
|  | What is the highest level of education you completed? | 1. No education 2. No formal education but read and write 3. Grade 1 to 8 4. Grade 9 to 12 5. Above grade 12 |  |
|  | What is the highest level of education your husband completed? | 1. No education 2. No formal education but read and write 3. Grade 1 to 8 4. Grade 9 to 12 5. Above grade 12 |  |
|  | What is your occupation? | 1. Government employee 2. Employee of private/NGO 3. Merchant 4. Housewife 5. Others(specify)________________ |  |
|  | What is your religion? | - 1. Protestant   2. Orthodox   3. Muslim   4. Catholic   5. Other(specify) ________________ |  |
|  | Residence | 1. Urban 2. Rural |  |
|  | Average family income per month (Eth. Birr} | - Mother_______________Birr/month - Husband __________birr/month - Additional income_________birr |  |

| **Part II: Obstetric history** | | | | |
| --- | --- | --- | --- | --- |
| **No** | | **Questions** | **Responses** | **Skip** |
|  | How many times you became pregnant? / | | _______________ |  |
|  | How many births have you given? | | _______________ |  |
|  | Have you had antenatal care follow up? | | - 1. No   2. Yes | If no, go to Q5 |
|  | If yes, number of visits | | ________ |  |
|  | Perinatal health care path? | | - 1. Start ANC with midwife, referred during ANC to Gynecologist   2. Start ANC with midwife, not referred   3. Start ANC with midwife, referred during labor to Gynecologist   4. No ANC, I came here during labor |  |
|  | Onset of labour | | - 1. Spontaneous   2. Induction   3. Elective Cd |  |
|  | What is route of current delivery? | | - 1. Vaginal   2. Instrumental   3. Caesarian delivery |  |
|  | Intervention during labor | | 1. No 2. Yes, no emergency intervention 3. Yes, emergency intervention |  |
|  | Antipain given during labor | | - 1. No requested   2. No medication received after requesting   3. Pain medication received after requesting |  |
|  | Time of delivery | | 1. Day    1. Night |  |
|  | Day of delivery | | 1. Weekend 2. Work day |  |
|  | Length of hospital stay | | _____________ day(s) |  |
|  | Duration of labor pain | | _____________ hours |  |
|  | Who has conducted the delivery?(qualification) | | ___________________________ |  |
|  | Adverse outcome of child | | 1. No 2. Yes |  |
|  | Obstetrics complication? | | - 1. No   2. Yes |  |
|  | Hospital admission of the mother? | | - 1. No   2. Yes |  |

**Part II Health Service Accessibility**

| 18. | Time to reach to this health facility | **--------------------------------------** |  |
| --- | --- | --- | --- |
| 19. | Availability of transport | 1. Yes 2. No |  |

**Part III: The eight domains with the questions developed for the maternity care**

| **General direction: Rate your experience from the choices at right for each question below.** | | | |
| --- | --- | --- | --- |
| **No** | **Domain and Items** | | **Rating** |
| **Dignity** | | | |
|  | I was treated respectfully during my experience. | | - Strongly disagree - Disagree - Neutral - Agree - Strongly agree |
|  | The physical examination and treatments were done in a way that respected my privacy. | | 1. Strongly disagree 2. Disagree 3. Neutral 4. Agree 5. Strongly agree |
|  | I was encouraged to ask questions about diseases, treatments and care. | | 1. Strongly disagree 2. Disagree 3. Neutral 4. Agree 5. Strongly agree |
|  | The health care provider gave me personal attention during history taking, physical examination and treatment. | | 1. Strongly disagree 2. Disagree 3. Neutral 4. Agree 5. Strongly agree |
|  | I was encouraged to discuss my concerns freely. | | 1. Strongly disagree 2. Disagree 3. Neutral 4. Agree 5. Strongly agree |
| **Autonomy** | | | |
|  | I was involved in making decisions for my treatment | | 1. Strongly disagree 2. Disagree 3. Neutral 4. Agree 5. Strongly agree |
|  | I received information about other types of treatments or tests. | | 1. Strongly disagree 2. Disagree 3. Neutral 4. Agree 5. Strongly agree |
|  | I had a choice to refuse examinations or treatments. | | 1. Strongly disagree 2. Disagree 3. Neutral 4. Agree 5. Strongly agree |
|  | I was asked for permission before starting testing or treatments. | | 1. Strongly disagree 2. Disagree 3. Neutral 4. Agree 5. Strongly agree |
| **Confidentiality** | | | |
|  | I was given the opportunity to speak privately with health care providers. | | 1. Strongly disagree 2. Disagree 3. Neutral 4. Agree 5. Strongly agree |
|  | My personal information was kept confidential. | | 1. Strongly disagree 2. Disagree 3. Neutral 4. Agree 5. Strongly agree |
|  | My medical record was kept confidential. | | 1. Strongly disagree 2. Disagree 3. Neutral 4. Agree 5. Strongly agree |
| **Communication** | | | |
|  | Things were explained clearly by my health care provider in way I could understand. | | 1. Strongly disagree 2. Disagree 3. Neutral 4. Agree 5. Strongly agree |
|  | I was encouraged to ask questions about my health problems, treatments and care. | | 1. Strongly disagree 2. Disagree 3. Neutral 4. Agree 5. Strongly agree |
|  | I was given enough time to ask questions about my health problems and treatments. | | 1. Strongly disagree 2. Disagree 3. Neutral 4. Agree 5. Strongly agree |
|  | My health care providers were responsive to my questions. | | 1. Strongly disagree 2. Disagree 3. Neutral 4. Agree 5. Strongly agree |
|  | My health care providers listened carefully to me. | | 1. Strongly disagree 2. Disagree 3. Neutral 4. Agree 5. Strongly agree |
| **Prompt attention** | | | |
|  | I received prompt attention at this health facility. | | 1. Strongly disagree 2. Disagree 3. Neutral 4. Agree 5. Strongly agree |
|  | The amount of time I waited before being attended to was acceptable. | | 1. Strongly disagree 2. Disagree 3. Neutral 4. Agree 5. Strongly agree |
|  | The travel time to this health service was acceptable. | | 1. Strongly disagree 2. Disagree 3. Neutral 4. Agree 5. Strongly agree |
| **Social support** | | | |
|  | It was easy to have family and friends visit me. | | 1. Strongly disagree 2. Disagree 3. Neutral 4. Agree 5. Strongly agree |
|  | I had the option of having an attendant stay with me during various stages of diagnosis and treatment. (This may not apply in special cases.) | | 1. Strongly disagree 2. Disagree 3. Neutral 4. Agree 5. Strongly agree |
| 1. . | My family and friends were able to bring care and services, such as food or other consumables | | 1. Strongly disagree 2. Disagree 3. Neutral 4. Agree 5. Strongly agree |
| **Choice** | | | |
|  | I had freedom in choosing this health care provider. | | 1. Strongly disagree 2. Disagree 3. Neutral 4. Agree 5. Strongly agree |
|  | I had the option to choose health care services I do not normally use. | | 1. Strongly disagree 2. Disagree 3. Neutral 4. Agree 5. Strongly agree |
|  | Continuity of care was provided by my individual health care provider. | | 1. Strongly disagree 2. Disagree 3. Neutral 4. Agree 5. Strongly agree |
| **Quality of basic amenities** | | | |
|  | | | |
|  | This health care facility, including toilets, examination room and linen, was clean. | | 1. Strongly disagree 2. Disagree 3. Neutral 4. Agree 5. Strongly agree |
|  | The amount of space provided was adequate. | | 1. Strongly disagree 2. Disagree 3. Neutral 4. Agree 5. Strongly agree |
|  | Health care personnel practised good hygiene (hands, clothes, linens, etc.). | | 1. Strongly disagree 2. Disagree 3. Neutral 4. Agree 5. Strongly agree |
|  | The department, bedroom, and bathroom were clean. | | 1. Strongly disagree 2. Disagree 3. Neutral 4. Agree 5. Strongly agree |
|  | Waiting areas and rooms had good air quality and ventilation. | | 1. Strongly disagree 2. Disagree 3. Neutral 4. Agree 5. Strongly agree |
| **Importance of Domains** | | | |
|  | Write the most important domains in delivery care | ________________________ | |

**Thank you for your participation!!**
