# Supplementary material for: Health system responsiveness and its associated factors for delivery care in public health facilities of West Arsi Zone, Oromia, Ethiopia
Source: PLoS One. 2026 Jan 7;21(1):e0340691. doi: 10.1371/journal.pone.0340691 (PMC12779144; doi:10.1371/journal.pone.0340691)
Supplement: S3 File — (DOCX) [file pone.0340691.s003.docx]

**Foormii Hayyama Hirmaannaa Qorannoo**

**Nagaa, Ani __________________________ jedhamaa.** Ani qorannoo gareen qorattoota Yuunivarsiitii Mada Walaabuu, Kaampaasii Shaashamannee, Godina ArsiiLixaa, Oromiyaa keessatti ilaalcha kenninsa tajaajila fayyaa fi haala walqabatan irratti geggeessuuf, odeeffannoo sassaabuuf bakka tajaajila kennaa jiru irratti hojjechaa jiru.

Kaayyoon qorannichaa tajaajila kenninsa fayyaa fi haala walqabatu kan buufata fayyaa mootummaa keessa jiru ilaalcha keessa galchuun akkaataa dhiyeenya isaa fi rakkoolee jiru xiinxaluudha. Qorannoo kana keessatti hirmaachuun akkaataa itti gafiiwwan qindaa’an deebisuu dandeessu irraa eegama, gafiiwwan kunneen karaa gaaffii afaanii (interview) ta’uun yeroo daqiiqaa 25 hanga 30 fudhachuu malu. Odeeffannoon isin irraa sassaabamu hundi iccitii keessatti eeggama, qorannoo kanaaf qofa kan oolu ta’a.

Foormiin kun maqaa keessan yookiin lakkoofsa beeksisaa hin gaafatu. Qorannoo kana keessatti hirmaachuun guutummaan guutuutti fedhii keessan irratti kan hundaa’e yoo ta’u, yeroo kamiyyuu hirmaachuu dhiisuufi keessaa ba’uu mirga ni qabdu. Hirmaachuu dhabuufi keessaa ba’uun adabbii yookaan tajaajila isin argattaniirratti miidhaa tokko illee hin geessisu.

**Hirmaattota qorannoo kabajamoo,**

Gaaffii kana guutuun keessan hirmaannaa keessan agarsiisa. Bu’aan qorannoo kunis rakkoolee kenninsa tajaajila fayyaa hawaasaaf buufata fayyaa mootummaa keessatti mul’atan adda baasuu irratti gargaara jedhamee eegama.

Odeeffannoon isin kennitan qofaaf kan ooluudha. Sirni kun iccitii irratti hundaa’ee tajaajila qorannichaaf ooluudha. Hirmaachuun fedhii keessan irratti hundaa’e, yeroo kamiyyuu hirmaachuu dhiisuu ni dandeessu. Hirmaachuu dhiisuufi keessaa ba’uun tajaajila isin argattu irraa miidhaa tokko illee hin geessisu.

**Odeeffannoo dabalataa yoo barbaaddan, lakkoofsa bilbilaa kana irratti nu quunnamaa:**
– Maqaa qorataa olaanaa: **Nagessoo Gabayyoo**
– Lakkoofsa bilbilaa: **0937335386**

Akka yeroo keessan kennitanii gaaffilee deebistan isin galateeffanna.

**Qorannoo kana keessatti hirmaachuu ni feeta?**
☐ Eeyyen, itti fufa. **Mallattoo_________________ Guyyaa________**
☐ Lakki, Galatoomi!

**Maqaa nama odeeffannoo sassaabu: _______________________________**
**Mallattoo__________________ Guyyaa________**
**Guyyaa ____________ Sa’aatii eegale ____________ Xumurame ____________**

# **Kutaa Lamaffaa: Foormii Odeeffannoo itti Guuramuu**

| Kutaa I – Amaloota Hawaasummaa fi Demografii | | | |
| --- | --- | --- | --- |
| **T.L** | **Gaafii** | **Deebii** | **Utaalii to** |
|  | Koodii/MRN | _________________ |  |
|  | Umrii kee meeqa? | _________________ (Woggaa) |  |
|  | Haala heeruma kee amma jiru maal? | 1. Kan hin heerumne 2. Heerume 3. Kan biraa (ibsi_____________________________ |  |
|  | Sadarkaa barnootaa ol’aanaa ati xumurte maal? | 1. Barnoota hin barannee 2. Barnoota sirrii hin qabne garuu dubbisuu fi barreessuu danda’a 3. Kutaa 1 hanga 8 4. Kutaa 9 hanga 12 5. kutaa 12 ol |  |
|  | Sadarkaa barnootaa ol’aanaa abban manaa kee xumuree maal? | 1. Barnoota hin barannee 2. Barnoota idilee hin qabuu garuu dubbisuu fi barreessuu danda’a 3. Kutaa 1 hanga 8 4. Kutaa 9 hanga 12 5. kutaa 12 ol |  |
|  | Hojii kee maal? | 1. Hojjetaa mootummaa 2. Hojjetaa dhaabbata dhuunfaa ykn NGO 3. Daldalaa 4. Haadha manaa 5. Kan biraa (ibsi) ________________ |  |
|  | What is your religion? | 1. Pirootestaantii 2. Ortodoksi 3. Musliima 4. Katoliki 5. Kaan (ibsi)_____________ |  |
|  | Teessoo jireenya kee | 1. Magaalaa 2. Baadiyyaa |  |
|  | Galii maatii giddu galeessa kan wagaa tokko meeqa? | ------------------------------------ |  |

| **Kutaa II: Seenaa Ulfaa fi Dhaloota** | | | | |
| --- | --- | --- | --- | --- |
| T.L | | Gaafii | Deebii | Utaalii |
|  | Yeroo meeqa ulfaa turte? | | _______________ |  |
|  | Yeroo meeqa dessee? | | _______________ |  |
|  | Tajaajila hordoffii ulfaa (ANC) argatteettaa? | | 0. Lakki 1. Eeyyee | Yoo laki ta’ee gara gaaffii 5tti deemii |
|  | Yoo eeyyee ta’e, yeroo daawwii meeqa? | | ________ |  |
|  | Daandii tajaajila fayyaa yeroo ulfaa? | | 0. hordoffii ulfaa jalqabe, gara espeshallistii ulfaatitii ergame  1. hordoffii ulfaa jalqabe, hin ergamne  2. hordoffii ulfaa jalqabe, yeroo ciniinsuu gara espeshallistii ulfaatitii ergame  3. hordoffii ulfaa jalqabe, yeroo ciniinsuu dhufe |  |
|  | Akkamitiisii jalqabee ciniinsuu? | | 0. Ofumaan jalqabee  1. qoriichaanjalqabee  2. Opireshiinii seenee |  |
|  | Mala ittiin amma dhalte maal? | | 0. karaa umamaan  1. Opireshiinii seenee  2. meeshaa fayyadmanii dahee |  |
|  | Yeroo ciniinsuu keessa tarkaanfii fudhatameera? | | 1. Lakki 2. Eeyyee, garuu tarkaanfii ariifachiisaa miti 3. Eeyyee, tarkaanfii ariifachiisaa fudhatame |  |
|  | Yeroo ciniinsuu qoricha dhukubiidhafi (antipain) siif laatameeraa? | | 0. Hin gaafanne  1. Gaafadhe, garuu hin arganne  2. Gaafadhe, erga gaafadheen booda argadhe |  |
|  | Yeroo dhalootaa (sa’aatii) | | 0. Guyyaa  1. Halkan |  |
|  | Guyyaa dessee | | 1. Sanbata/dilbata 2. Guyyaa hojii |  |
|  | Yeroo hospitaalaa turte | | _____________ sa’aatii |  |
|  | Yeroo ciniinsuu irrati turte | | _____________ sa’aatii |  |
|  | Eenyutu sii desiissee? (Ogummaa) | | ___________________________ |  |
|  | Miidhaa daa’imman irratti mul’ate? | | 0. Lakki  1. Eeyyee |  |
|  | Rakkoo ulfaa (obstetric complication) mudate? | | 0. Lakki  1. Eeyyee |  |
|  | Ulfa kana irrati hospitaala seente turte? | | 0. Lakki  1. Eeyyee |  |

**Kutaa III: Dhaqqabummaa Tajaajila Fayyaa**

| 1. | Gara buufata fayyaa kanaatti ga’uufi sa’aatii hangami siti fudhataa | **--------------------------------------** |  |
| --- | --- | --- | --- |
|  | Argama geejjibaa | 0.Jiraa  1.Hin jiruu |  |

**Kutaa IV: Gaaffilee Gosa Saddeetii Tajaajila Haadholiif Qophaa’an**

| **Qajeelfama Waliigalaa: Muuxannoo kee, gaaffilee armaan gadiitiif filannoo mirga jiruun madaali.** | | | |
| --- | --- | --- | --- |
| **Lak** | **Kutaa fi Gaaffilee** | | **Madaallii** |
| **Kabaja** | | | |
|  | Yeroo ani achi turetti kabajaan naaf kenname.. | | 1. Sirritti walii hin galuu 2. walii hin galuu 3. Giddu galeessa 4. Walii galaa 5. Siritii walii galaa |
|  | Muummee qaamaa fi yaaliin naaf taasifame, iccitii koo kabajuun gaggeeffame. | | 1. Sirritti walii hin galuu 2. walii hin galuu 3. Giddu galeessa 4. Walii galaa 5. Siritii walii galaa |
|  | Gaaffiiwwan waa'ee dhukkubaa, yaalii fi kunuunsa irratti gaafachuuf na jajjabeessan. | | - 1. Sirritti walii hin galuu   2. walii hin galuu   3. Giddu galeessa   4. Walii gala   5. Siritii walii galaa |
|  | Ogeessi fayyaa yeroo seenaa na gaafachaa, qorannoo qaamaa fi yaalii taasisaa turanitti xiyyeeffannaa dhuunfaa naaf kennuun na tajaajile. | | 1. Sirritti walii hin galuu 2. walii hin galuu 3. Giddu galeessa 4. Walii galaa 5. Siritii walii galaa |
|  | Yaaddoo fi komii koo bilisaan akka ibsu fi mari’achuuf na jajjabeessan. | | 1. Sirritti walii hin galuu 2. walii hin galuu 3. Giddu galeessa 4. Walii galaa 5. Siritii walii galaa |
| **Bilisummaa murteefannaa dhuunfaa** | | | |
|  | Murtee yaalii naaf taasifamu keessatti ani qooda qabaachuuf affeerame. | | 1. Sirritti walii hin galuu 2. walii hin galuu 3. Giddu galeessa 4. Walii galaa 5. Siritii walii galaa |
|  | Odeeffannoo waa'ee gosa yaalii yookiin qorannoo biraa argadhe. | | 1. Sirritti walii hin galuu 2. walii hin galuu 3. Giddu galeessa 4. Walii galaa 5. Siritii walii galaa |
|  | Qorannoo fi yaalii dhiisuuf mirgaafi filannoo qaba ture. | | 1. Sirritti walii hin galuu 2. walii hin galuu 3. Giddu galeessa 4. Walii galaa 5. Siritii walii galaa |
|  | Qorannoo fi yaalii jalqabuu dura eeyyama koo akka kennuuf na gaafatan. | | 1. Sirritti walii hin galuu 2. walii hin galuu 3. Giddu galeessa 4. Walii galaa 5. Siritii walii galaa |
| **Eeggannoo iccitii** | | | |
|  | Ogeessota fayyaa waliin iccitii ta’een haasa’uuf carraan naa kenname. | | 1. Sirritti walii hin galuu 2. walii hin galuu 3. Giddu galeessa 4. Walii galaa 5. Siritii walii galaa |
|  | Odeeffannoon kiyya dhuunfaa iccitii ta’ee ni eegame. | | 1. Sirritti walii hin galuu 2. walii hin galuu 3. Giddu galeessa 4. Walii galaa 5. Siritii walii galaa |
|  | Galmeen fayyaa koo iccitii ta’ee ni eegame. | | 1. Sirritti walii hin galuu 2. walii hin galuu 3. Giddu galeessa 4. Walii galaa 5. Siritii walii galaa |
| **Qunnamtii** | | | |
|  | Ogeessi fayyaa odeeffannoo ani hubachuu danda’u fi ifa ta’eedhaan naaf ibse. | | 1. Sirritti walii hin galuu 2. walii hin galuu 3. Giddu galeessa 4. Walii galaa 5. Siritii walii galaa |
|  | Waa’ee rakkoo fayyaa, yaalii fi kunuunsa koo irratti gaaffii dhiyeessuu akka danda’uuf na jajjabeessan. | | 1. Sirritti walii hin galuu 2. walii hin galuu 3. Giddu galeessa 4. Walii galaa 5. Siritii walii galaa |
|  | Rakkoo fayyaa fi yaalii koo irratti gaaffii dhiyeessuu danda’uuf yeroo gahaan naa kenname. | | 1. Sirritti walii hin galuu 2. walii hin galuu 3. Giddu galeessa 4. Walii gala 5. Siritii walii galaa |
|  | Ogeeyyonni fayyaa gaaffiiwwan koo sirnaan deebisanii fi naaf ibsan. | | 1. Sirritti walii hin galuu 2. walii hin galuu 3. Giddu galeessa 4. Walii galaa 5. Siritii walii galaa |
|  | Ogeeyyonni fayyaa wantoota ani jedhe sirriitti na dhaggeeffatan. | | 1. Sirritti walii hin galuu 2. walii hin galuu 3. Giddu galeessa 4. Walii galaa 5. Siritii walii galaa |
| **Xiyyeeffannaa hatattamaa** | | | |
|  | Xiyyeeffannaa ariifachiisaa mana yaalaa kana keessatti naaf kennan. | | 1. Sirritti walii hin galuu 2. walii hin galuu 3. Giddu galeessa 4. Walii galaa 5. Siritii walii galaa |
|  | Yeroo tajaajila eeguuf ture gahaa fi fudhatama qabu ture. | | 1. Strongly disagree 2. Sirritti walii hin galuu 3. walii hin galuu 4. Giddu galeessa 5. Walii galaa 6. Siritii walii galaa |
|  | Yeroo gara tajaajila fayyaa kanaatti imaluun fudhate fudhatama qaba ture. | | 1. Strongly disagree 2. Sirritti walii hin galuu 3. walii hin galuu 4. Giddu galeessa 5. Walii galaa 6. Siritii walii galaa |
| **Deeggarsa maatii fi hiriyoota** | | | |
|  | Daawwannaa maatii fi hiriyootaa argachuun naaf salphaa ture. | | 1. Sirritti walii hin galuu 2. walii hin galuu 3. Giddu galeessa 4. Walii galaa 5. Siritii walii galaa |
|  | Yeroo gosa adda addaa qorannoo fi yaaliitti nama na tajaajilu na bira akka tursisuuf filannoon naa kennameera. (Kun haala addaa keessatti kan hin hojjanne ta’uu danda’a.) | | 1. Sirritti walii hin galuu 2. walii hin galuu 3. Giddu galeessa 4. Walii galaa 5. Siritii walii galaa |
| 1. . | Maatii fi hiriyoonni koo nyaataa fi meeshaalee itti fayyadamoo biroo akkasumas tajaajila naaf fiduu danda’an. | | 1. Sirritti walii hin galuu 2. walii hin galuu 3. Giddu galeessa 4. Walii galaa 5. Siritii walii galaa |
| **Filannoo** | | | |
|  | Filannoo bilisaa ogeessa fayyaa kana filachuu keessatti qaba ture. | | 1. Sirritti walii hin galuu 2. walii hin galuu 3. Giddu galeessa 4. Walii galaa 5. Siritii walii galaa |
|  | Tajaajila fayyaa yeroo hunda hin fayyadamne filachuuf filannoo qaba ture. | | 1. Sirritti walii hin galuu 2. walii hin galuu 3. Giddu galeessa 4. Walii galaa 5. Siritii walii galaa |
|  | Tajaajilli fayyaa itti fufinsa qabu ogeessa fayyaa kiyyaan naaf kennamaa ture. | | 1. Sirritti walii hin galuu 2. walii hin galuu 3. Giddu galeessa 4. Walii galaa 5. Siritii walii galaa |
| **Qulqullina tajaajiloota bu’uuraa** | | | |
|  | | | |
|  | Tajaajilli fayyaa kun, mana fincaanii, kutaa qorannoo fi kutaa ciisichaa dabalatee qulqullina qaba ture. | | 1. Sirritti walii hin galuu 2. walii hin galuu 3. Giddu galeessa 4. Walii galaa 5. Siritii walii galaa |
|  | Bal’inni iddoo naaf kenname gahaa fi mijataa ture. | | 1. Sirritti walii hin galuu 2. walii hin galuu 3. Giddu galeessa 4. Walii galaa 5. Siritii walii galaa |
|  | Hojjettoonni fayyaa qulqullina harka, uffata, fi meeshaalee fayyaa biroorratti xiyyeeffannoo kennuun sirnaan eeguu turan. | | 1. Sirritti walii hin galuu 2. walii hin galuu 3. Giddu galeessa 4. Walii galaa 5. Siritii walii galaa |
|  | Kutaan tajaajilaa, bakka ciisichaa fi mana fincaanii qulqullina gaarii qabu ture. | | 1. Sirritti walii hin galuu 2. walii hin galuu 3. Giddu galeessa 4. Walii galaa 5. Siritii walii galaa |
|  | Bakki eegumsaafi kutaan tajaajilaa qilleensa qulqulluu fi qilleensa ittiin naanna’u gahaa qaba ture. | | 1. Sirritti walii hin galuu 2. walii hin galuu 3. Giddu galeessa 4. Walii galaa 5. Siritii walii galaa |
| **Barbaachisummaa Kutaa Gurguddoo Domeniiwwan** | | | |
|  | Domenii issa kamtuu faayidaa gudda qabaa | ________________________ | |

**Hirmaannaa keessaniif hedduu galatoomaa!**
