# Supplementary material for: Health system responsiveness and its associated factors for delivery care in public health facilities of West Arsi Zone, Oromia, Ethiopia
Source: PLoS One. 2026 Jan 7;21(1):e0340691. doi: 10.1371/journal.pone.0340691 (PMC12779144; doi:10.1371/journal.pone.0340691)
Supplement: Attachment — Submitted filename: PONE-D-25-48434_reviewer - Abdulmalik Abubakar.docx [file pone.0340691.s004.docx]

**Health System Responsiveness and its associated factors for Delivery Care in Public Health Facilities of West Arsi Zone, Oromia, Ethiopia**

**Major Methodological & Reporting Issues**

| **Location in manuscript** | **Issue** | **Action Required** |
| --- | --- | --- |
| Abstract & Objective | The Objective states the study period is October 2024 to June 2025. Given the current date (October 2025), this suggests the study is either still ongoing or the reporting dates are incorrect for a completed study. | Correct the dates in the Abstract and Methods to reflect the actual time the data was collected and analyzed. |
| Abstract: Methods | The description of the outcome (HSR) is inadequate. It is referred to only as "HSR" without specifying if it's the overall score, the proportion of responsive/unresponsive classification, or a mean/median score. | Clearly state how the HSR outcome variable was measured, calculated, and categorized (e.g., "HSR was categorized as 'responsive' or 'unresponsive' based on a median split of the overall score"). |
| Results: Regression (Abstract & Main Text) | The manuscript must clearly report the adjusted odds ratios (AORs) and 95% Confidence Intervals (CIs) for the associated factors (e.g., in the Findings section of the Abstract). Currently, it only mentions that four factors were associated, which is insufficient. | Provide the specific AORs and CIs in the Abstract and ensure the full regression output is available in the Main Text or a supplementary table. |

**Minor Typos and Clarity**

| **Location** | **Original Text** | **Suggested Correction/Improvement** |
| --- | --- | --- |
| Full Title | Health System Responsiveness and its associated factors for Delivery Care in Public Health Facilities of West Arsi Zone, Oromia, Ethiopia | Health System Responsiveness and its **Associated** Factors for **Delivery Care** in Public Health Facilities of West Arsi Zone, Oromia, Ethiopia (Use title case for major words). |
| Abstract: Background | guiding future strategies However, there remains a scarcity... | guiding future strategies. **However,** there remains a scarcity... (Missing period and capital letter). |
| Abstract: Methods | Health facility based cross-sectional stud... | Health facility-based cross-sectional **study**... (Incomplete word). |
| Abstract: Findings | The overall magnitude of health system responsiveness was found to be 58.6%... | The overall **level** of health system responsiveness was found to be **58.6%**... (Use "level" instead of "magnitude" for a percentage; this phrasing is stronger). |
| Keywords | Health system responsiveness, delivery care, West Arsi Zone | Add "associated factors" or "cross-sectional" to improve searchability (e.g., Health System Responsiveness; Delivery Care; Associated Factors; Ethiopia). |
