# Supplementary material for: Health system responsiveness and its associated factors for delivery care in public health facilities of West Arsi Zone, Oromia, Ethiopia
Source: PLoS One. 2026 Jan 7;21(1):e0340691. doi: 10.1371/journal.pone.0340691 (PMC12779144; doi:10.1371/journal.pone.0340691)
Supplement: Attachment — Submitted filename: REVIWERS COMMENTS.docx [file pone.0340691.s005.docx]

The commentary focuses heavily on the role of technology in achieving UHC but does not sufficiently address the importance of integrating technology with other health system strengthening efforts, such as improving primary healthcare, training healthcare workers, and addressing social determinants of health.

While technology plays a transformative role in achieving UHC, it must be integrated with broader health system strengthening efforts to ensure sustainable impact. Strengthening primary healthcare systems remains a cornerstone of UHC, providing a foundation for preventive and curative care. Digital solutions should complement these efforts by enhancing service delivery, streamlining patient referrals, and supporting remote consultations. Moreover, investing in healthcare workforce training is critical to ensure that digital tools are effectively utilized. Capacity-building initiatives should focus on equipping healthcare providers with the necessary digital literacy skills to maximize the benefits of AI-driven diagnostics, telemedicine, and electronic health records. Additionally, addressing social determinants of health—such as poverty, education, and access to clean water—is essential for achieving equitable health outcomes. Without a holistic approach that combines technological innovation with systemic health improvements, the potential of digital health solutions to drive meaningful progress in UHC may be limited.

These applications demonstrate how AI can contribute to achieving UHC by enhancing healthcare accessibility, efficiency, and quality. By leveraging AI-driven innovations, health systems can improve early diagnosis, optimize resource allocation, and provide personalized care, ultimately reducing disparities and improving patient outcomes.

Incorporating AI within a broader digital health strategy will be critical in ensuring that technological advancements translate into equitable healthcare solutions. Further discussion on regulatory challenges, data privacy concerns, and implementation barriers would be beneficial to ensure responsible AI deployment in healthcare.

**Integrating Technology with Health System Strengthening Efforts**

While technology plays a critical role in advancing Universal Health Coverage (UHC), its full potential can only be realized when integrated with broader health system strengthening initiatives. **Primary healthcare (PHC)** remains the cornerstone of achieving UHC, as emphasized by the World Health Organization. Digital innovations should complement efforts to enhance PHC by improving service delivery, enabling better coordination of care, and expanding access to essential medicines and diagnostics in underserved areas.

**Training healthcare workers** is another vital component of health system strengthening. Technology can support capacity building through online training platforms, virtual simulations, and mobile-based learning tools, enabling healthcare providers to acquire new skills and stay updated on best practices. For example, mobile learning applications have been used successfully in Kenya to train community health workers on maternal and child health interventions, leading to improved service delivery and patient outcomes.

Addressing **social determinants of health** is equally important in creating equitable healthcare systems. Technology can facilitate data collection and analysis to identify social factors—such as poverty, education, housing, and nutrition—that impact health outcomes. For instance, geographic information systems (GIS) have been used in South Africa to map underserved areas and target interventions more effectively. Additionally, digital platforms can empower communities by providing health education tailored to local contexts and languages.

By integrating technology with these foundational elements of health systems—PHC strengthening, workforce development, and addressing social determinants—countries can ensure that digital innovations contribute not only to improved efficiency but also to equitable access and quality care for all.

Integrating Technology with Health System Strengthening Efforts

While technology plays a critical role in advancing Universal Health Coverage (UHC), its full potential can only be realized when integrated with broader health system strengthening initiatives. Primary healthcare (PHC) remains the cornerstone of achieving UHC, as emphasized by the World Health Organization. Digital innovations should complement efforts to enhance PHC by improving service delivery, enabling better coordination of care, and expanding access to essential medicines and diagnostics in underserved areas.

Training healthcare workers is another vital component of health system strengthening. Technology can support capacity building through online training platforms, virtual simulations, and mobile-based learning tools, enabling healthcare providers to acquire new skills and stay updated on best practices. For example, mobile learning applications have been used successfully in Kenya to train community health workers on maternal and child health interventions, leading to improved service delivery and patient outcomes.

Addressing social determinants of health is equally important in creating equitable healthcare systems. Technology can facilitate data collection and analysis to identify social factors—such as poverty, education, housing, and nutrition—that impact health outcomes. For instance, geographic information systems (GIS) have been used in South Africa to map underserved areas and target interventions more effectively. Additionally, digital platforms can empower communities by providing health education tailored to local contexts and languages.

Moreover, the integration of technology in healthcare must be accompanied by robust data privacy and security measures. As digital health systems collect and store sensitive patient information, it is crucial to protect this data from unauthorized access, cyberattacks, and misuse. Governments and healthcare organizations should implement strong cybersecurity protocols, data encryption techniques, and access controls to safeguard patient privacy and maintain trust in digital health systems. Additionally, ethical considerations surrounding the use of technology in healthcare should be addressed, including issues such as algorithmic bias, data ownership, and the potential for discrimination. Finally, promoting interoperability and data standards is essential to ensure seamless data exchange and coordination of care across different digital health systems. Governments and organizations can promote interoperability by adopting standardized data formats, protocols, and application programming interfaces (APIs), which allow different systems to communicate and share information securely and efficiently.

By integrating technology with these foundational elements of health systems—PHC strengthening, workforce development, addressing social determinants, ensuring data privacy and security, and promoting interoperability—countries can ensure that digital innovations contribute not only to improved efficiency but also to equitable access and quality care for all.

By including these additional points, the manuscript will provide a more nuanced and comprehensive perspective on the challenges and opportunities of using technology to advance UHC in Africa. Remember to back up your points with evidence and examples whenever possible.

**Comment 5:** Limited Discussion on Sustainability:

The commentary briefly mentions the need for context-specific solutions but does not fully explore the sustainability of technology-based interventions. A discussion on how to ensure the long-term viability and scalability of these solutions would be valuable.

Transformative Potential of Technology in Healthcare

Technological advancements, particularly in the fintech and mobile sectors, offer enormous potential for achieving UHC in Africa. The fintech sector facilitates financial inclusion for previously unbankable groups, paving the way for affordable healthcare (8). Digital health innovations like telemedicine and mHealth have expanded affordable access to care, making it easier for people of all demographics to obtain essential medical care. Mobile technology is transforming the African economy by enabling those without smartphones or traditional bank accounts to access healthcare services through innovative mobile money solutions, such as M-Pesa in Kenya, which has become a dominant payment method driving innovation in commerce and healthcare (11).

Data-driven healthcare solutions optimize data management and patient-provider interaction, reducing inefficiencies in healthcare delivery. Real-time data improves care quality, transparency, and cost-effectiveness, ultimately benefiting community health (12). Digital health financing systems, like CarePay in Kenya, enable underprivileged populations to access affordable health insurance coverage, closing healthcare access gaps (13). Developing an innovation ecosystem for UHC, involving cooperation between the private sector and other stakeholders, can improve healthcare accessibility through creative service delivery, health technology, and capacity building (7).

However, to ensure the long-term viability and scalability of technology-based interventions, it is crucial to address issues of sustainability. This includes investing in robust infrastructure (reliable electricity, internet connectivity) and building local capacity for maintaining and updating digital health systems. Financial sustainability can be enhanced through innovative financing models, such as blended finance and public-private partnerships, that mobilize resources from both domestic and international sources. Additionally, community engagement and ownership are essential for ensuring that technology-based interventions are aligned with local needs and priorities, promoting their long-term adoption and utilization. By addressing these sustainability challenges, technology can truly transform healthcare delivery and contribute to lasting improvements in health outcomes across Africa.

**Comment 6:** Ethical Considerations:

While privacy and data security are mentioned, the commentary could expand on the ethical implications of using technology in healthcare, such as algorithmic bias in AI, the digital divide, and the potential for technology to reinforce existing power imbalances.

Here's the revised "Transformative Potential of Technology in Healthcare" section with the added paragraph:

"Technological advancements, particularly in the fintech and mobile sectors, offer enormous potential for achieving UHC in Africa. The fintech sector facilitates financial inclusion for previously unbankable groups, paving the way for affordable healthcare (8). Digital health innovations like telemedicine and mHealth have expanded affordable access to care, making it easier for people of all demographics to obtain essential medical care. Mobile technology is transforming the African economy by enabling those without smartphones or traditional bank accounts to access healthcare services through innovative mobile money solutions, such as M-Pesa in Kenya, which has become a dominant payment method driving innovation in commerce and healthcare (11).

Data-driven healthcare solutions optimize data management and patient-provider interaction, reducing inefficiencies in healthcare delivery. Real-time data improves care quality, transparency, and cost-effectiveness, ultimately benefiting community health (12). Digital health financing systems, like CarePay in Kenya, enable underprivileged populations to access affordable health insurance coverage, closing healthcare access gaps (13). Developing an innovation ecosystem for UHC, involving cooperation between the private sector and other stakeholders, can improve healthcare accessibility through creative service delivery, health technology, and capacity building (7).

However, it is essential to acknowledge the ethical implications of technology in healthcare. Algorithmic bias in AI systems can perpetuate existing inequalities, leading to disparities in diagnosis and treatment. The digital divide can exacerbate exclusion, as marginalized communities may lack access to the technologies and digital literacy skills needed to benefit from digital health solutions. Additionally, technology has the potential to reinforce existing power imbalances, with wealthier or more educated individuals benefiting disproportionately. To address these ethical challenges, it is crucial to prioritize equity, transparency, and accountability in the design and implementation of technology-based healthcare interventions. This includes promoting digital inclusion, ensuring that algorithms are free from bias, and empowering communities to participate in shaping the development and deployment of digital health solutions."

Include Stakeholder Perspectives:

Consider including perspectives from key stakeholders, such as healthcare providers, patients, and policymakers, to provide a more holistic view of the challenges and opportunities.

Here's the revised "Transformative Potential of Technology in Healthcare" section:

"Technological advancements, particularly in the fintech and mobile sectors, offer enormous potential for achieving UHC in Africa. The fintech sector facilitates financial inclusion for previously unbankable groups, paving the way for affordable healthcare (8). Digital health innovations like telemedicine and mHealth have expanded affordable access to care, making it easier for people of all demographics to obtain essential medical care. Mobile technology is transforming the African economy by enabling those without smartphones or traditional bank accounts to access healthcare services through innovative mobile money solutions, such as M-Pesa in Kenya, which has become a dominant payment method driving innovation in commerce and healthcare (11).

Data-driven healthcare solutions optimize data management and patient-provider interaction, reducing inefficiencies in healthcare delivery. Real-time data improves care quality, transparency, and cost-effectiveness, ultimately benefiting community health (12). Digital health financing systems, like CarePay in Kenya, enable underprivileged populations to access affordable health insurance coverage, closing healthcare access gaps (13). Developing an innovation ecosystem for UHC, involving cooperation between the private sector and other stakeholders, can improve healthcare accessibility through creative service delivery, health technology, and capacity building (7).

It is critical to consider the perspectives of various stakeholders when evaluating technology's transformative potential. Healthcare providers often highlight the need for user-friendly technologies that seamlessly integrate into their workflows and reduce administrative burdens. Patients emphasize the importance of accessible and affordable digital health solutions that empower them to manage their health and access timely care. Policymakers stress the need for evidence-based interventions that align with national health priorities and contribute to UHC goals. By incorporating these stakeholder perspectives, we can ensure that technology is harnessed in a way that truly benefits all members of society and promotes equitable access to quality healthcare."

Reviewer 2: It was a pleasure to review your research, this is an interesting and valuable topic. It has the potential to be published in the future, after minor revisions, including:

- The manuscript provides a comprehensive overview of the role of technology in advancing Universal Health Coverage (UHC) in Africa. However, it lacks depth in analyzing the socio-cultural and political barriers that may hinder the adoption of these technologies. For instance, the manuscript could explore how cultural resistance to digital health tools or political instability in certain regions might affect implementation.
