# Supplementary material for: Health system responsiveness and its associated factors for delivery care in public health facilities of West Arsi Zone, Oromia, Ethiopia
Source: PLoS One. 2026 Jan 7;21(1):e0340691. doi: 10.1371/journal.pone.0340691 (PMC12779144; doi:10.1371/journal.pone.0340691)
Supplement: Attachment — Submitted filename: Response to Reviewers.docx [file pone.0340691.s006.docx]

**A Rebuttal Letter**

We would like to thank journal editors, both reviewers for their insightful comments and we have refined the manuscript as per their comments and presented it as follows:

**Journal Requirements**

1. When submitting your revision, we need you to address these additional requirements.

Please ensure that your manuscript meets PLOS ONE's style requirements, including those for file naming. The PLOS ONE style templates can be found at

https://journals.plos.org/plosone/s/file?id=wjVg/PLOSOne_formatting_sample_main_body.pdf and

<https://journals.plos.org/plosone/s/file?id=ba62/PLOSOne_formatting_sample_title_authors_affiliations.pdf>

**Response:** We have ensured that all documents fully comply with the journal’s submission requirements.

2. Please include a complete copy of PLOS’ questionnaire on inclusivity in global research in your revised manuscript. Our policy for research in this area aims to improve transparency in the reporting of research performed outside of researchers’ own country or community. The policy applies to researchers who have travelled to a different country to conduct research, research with Indigenous populations or their lands, and research on cultural artefacts. The questionnaire can also be requested at the journal’s discretion for any other submissions, even if these conditions are not met. Please find more information on the policy and a link to download a blank copy of the questionnaire here: https://journals.plos.org/plosone/s/best-practices-in-research-reporting. Please upload a completed version of your questionnaire as Supporting Information when you resubmit your manuscript.

**Response:** We have uploaded a copy of PLOS’ questionnaire on inclusivity in global research. Besides, we have added information in the methods section requested in the questionnaire.

3. Please amend either the abstract on the online submission form (via Edit Submission) or the abstract in the manuscript so that they are identical.

**Response:** We have revised the abstract to ensure that the version in the online submission form matches the one in the manuscript.

4. We note that this data set consists of interview transcripts. Can you please confirm that all participants gave consent for interview transcript to be published?

If they DID provide consent for these transcripts to be published, please also confirm that the transcripts do not contain any potentially identifying information (or let us know if the participants consented to having their personal details published and made publicly available). We consider the following details to be identifying information:

- Names, nicknames, and initials

- Age more specific than round numbers

- GPS coordinates, physical addresses, IP addresses, email addresses

- Information in small sample sizes (e.g. 40 students from X class in X year at X university)

- Specific dates (e.g. visit dates, interview dates)

- ID numbers

Or, if the participants DID NOT provide consent for these transcripts to be published:

- Provide a de-identified version of the data or excerpts of interview responses

- Provide information regarding how these transcripts can be accessed by researchers who meet the criteria for access to confidential data, including:

a) the grounds for restriction

b) the name of the ethics committee, Institutional Review Board, or third-party organization that is imposing sharing restrictions on the data

c) a non-author, institutional point of contact that is able to field data access queries, in the interest of maintaining long-term data accessibility.

d) Any relevant data set names, URLs, DOIs, etc. that an independent researcher would need in order to request your minimal data set.

For further information on sharing data that contains sensitive participant information, please see: https://journals.plos.org/plosone/s/data-availability#loc-human-research-participant-data-and-other-sensitive-data

If there are ethical, legal, or third-party restrictions upon your dataset, you must provide all of the following details (https://journals.plos.org/plosone/s/data-availability#loc-acceptable-data-access-restrictions):

a) A complete description of the dataset

b) The nature of the restrictions upon the data (ethical, legal, or owned by a third party) and the reasoning behind them

c) The full name of the body imposing the restrictions upon your dataset (ethics committee, institution, data access committee, etc)

d) If the data are owned by a third party, confirmation of whether the authors received any special privileges in accessing the data that other researchers would not have

e) Direct, non-author contact information (preferably email) for the body imposing the restrictions upon the data, to which data access requests can be sent.

**Response:** We have removed all identifying information from the data set. Besides, the full transcripts are subject to restricted access for the following reasons:

a) Protection of participant privacy, as participants did not consent to public release of full transcripts.

b) Data-sharing restrictions imposed by the Research Review and Ethics Committee of Madda Walabu University, Shashemene Campus, which approved the study protocol.

c) A non-author institutional contact person for data access inquiries is:

**Mr. Musa Kumbi**

Research, Community Engagement, and Technology Transfer Coordinator

College of Health Sciences, Madda Walabu University, Shashemene Campus

Email: [mukumbi2@gmail.com](mailto:mukumbi2@gmail.com)

He is responsible for reviewing and responding to qualified requests for access to the confidential data.

Researchers who meet the eligibility criteria may request the minimal de-identified dataset through the institution. The dataset is stored under the following reference:

**Dataset Name:** Final Cleaned HSR Data

This process ensures long-term accessibility of the dataset for qualified researchers while maintaining strict protection of participant confidentiality.

5. If the reviewer comments include a recommendation to cite specific previously published works, please review and evaluate these publications to determine whether they are relevant and should be cited. There is no requirement to cite these works unless the editor has indicated otherwise.

**Response:** There are no reviewer’s recommendation to cite specific previously published works.

**Reviewer # 1:**

**Comment:** The Objective states the study period is October 2024 to June 2025. Given the current date (October 2025), this suggests the study is either still ongoing or the reporting dates are incorrect for a completed study. Correct the dates in the Abstract and Methods to reflect the actual time the data was collected and analyzed.

**Response:** We have refined as per the comment given.

**Comment:** The description of the outcome (HSR) is inadequate. It is referred to only as "HSR" without specifying if it's the overall score, the proportion of responsive/unresponsive classification, or a mean/median score. Clearly state how the HSR outcome variable was measured, calculated, and categorized (e.g., "HSR was categorized as 'responsive' or 'unresponsive' based on a median split of the overall score").

**Response:** We have refined as per the comment given.

**Comment:** The manuscript must clearly report the adjusted odds ratios (AORs) and 95% Confidence Intervals (CIs) for the associated factors (e.g., in the Findings section of the Abstract). Currently, it only mentions that four factors were associated, which is insufficient.	Provide the specific AORs and CIs in the Abstract and ensure the full regression output is available in the Main Text or a supplementary table.

**Response:** We would like to clarify for the reviewer that we have already written adjusted odds ratios (AORs) and 95% Confidence Intervals (CIs) for the associated factors. In the result section, we have clearly reported full logistic regression table in the result section, specifically in Table 4. It’s clear that in the abstract section, major findings need to be reported as it is the summary of all research work.

**Comment:** Health System Responsiveness and its associated factors for Delivery Care in Public Health Facilities of West Arsi Zone, Oromia, Ethiopia Health System Responsiveness and its **Associated** Factors for **Delivery Care** in Public Health Facilities of West Arsi Zone, Oromia, Ethiopia (Use title case for major words).

**Response:** We would like to kindly clarify that as per the PLos one title guideline, a title has to be concise and written in sentence case. Here is the title “Health system responsiveness and its associated factors for Delivery Care in Public Health Facilities of West Arsi Zone, Oromia, Ethiopia.”

**Comment:** Abstract: Background….guiding future strategies However, there remains a scarcity... guiding future strategies. **However,** there remains a scarcity... (Missing period and capital letter).

**Response:** We have refined as per the comment given.

**Comment:** Abstract: Methods: Health facility based cross-sectional stud...Health facility-based cross-sectional **study**... (Incomplete word).

**Response:** We have refined as per the comment given.

**Comment:** Abstract: Findings: The overall magnitude of health system responsiveness was found to be 58.6%...	The overall **level** of health system responsiveness was found to be **58.6%**... (Use "level" instead of "magnitude" for a percentage; this phrasing is stronger).

**Response:** We have refined as per the comment given.

**Comment:** Keywords	Health system responsiveness, delivery care, West Arsi Zone Add "associated factors" or "cross-sectional" to improve searchability (e.g., Health System Responsiveness; Delivery Care; Associated Factors; Ethiopia).

**Response:** We have refined as per the comment given.

**Reviewer # 1:**

**Comment:** Abstract (pp. 10–11, lines 26–57)

The abstract summarizes the study well but could be more precise in reporting key findings. For example, clarify what “relatively higher than similar studies”

The phrase “crucial in impact health system responsiveness” (line 55) is unclear—please rephrase for clarity.

May include the exact domains assessed (if it will not impact word count) in the abstract for completeness.

**Response:** We have refined as per the comment given.

**Introduction**

**Comment:** The introduction provides extensive global context but could be condensed. Lines 70–81 on SDGs and Agenda 2063 can be summarized to focus more on Ethiopia-specific gaps.

**Response:** We have refined as per the comment given.

**Comment:** The definition of HSR is clear, but the manuscript could benefit from a brief explanation of why each of the eight domains (e.g., dignity, autonomy, choice) is critical in delivery care. where should this be addressed.

**Response:** We have included this description in the measurement section under the methodology part.

**Comment:** Clearly articulate the novelty of this study compared to previous Ethiopian studies (e.g., inclusion of health centers, timing near HSTP-II conclusion).

**Response:** We have refined as per the comment given.

**Comment:** Include recent literature on health system responsiveness and respectful maternity care.

**Response:** We have included latest literature (Check reference no.8 & 9).

Methods (pp. 12–15, lines 103–193)

**Comment:** Sampling: Lines 116–130 describe sampling but lack detail on how systematic sampling was implemented. Clarify calculation of K and proportional allocation across facilities.

**Response:** We have refined as per the comment given.

**Comment:** Measurement: Lines 144–155—justify dichotomization using median and explain why WHO responsiveness tool was used without local adaptation. Provide Cronbach’s alpha values for each domain.

**Response:** We have refined as per the comment given and provided Cronbach’s alpha values for the respective domains.

**Comment:** Data Analysis: Lines 162–166—justify p<0.30 threshold for variable selection. Report VIF values explicitly.

**Response:** We have justified the rationale for using p-value <0.30 and also reported VIF values.

**Comment:** Include ethics approval number (line 178).

**Response:** We have included ethical approval number.

**Results (pp. 16–22, lines 194–303)**

**Comment:** Tables 1–3 (pp. 17–20) need improved formatting for clarity—align percentages and frequencies.

**Response:** We have improved formatting as per the comment given.

**Comment:** Figure 2 (p. 21) should include confidence intervals or error bars for domain scores.

**Response:** We have included confidence intervals/error bars for the respective domains.

**Comment:** Consider adding a summary table for domain-wise responsiveness percentages (lines 277–279).

**Response:** We have added a summary table for the respective domains

**Comment:** Report Hosmer-Lemeshow test results in the results section, not only in methods.

**Response:** We have reported it in the result section.

**Discussion (pp. 24–26, lines 321–395)**

**Comment:** Comparisons with other studies are useful but lack depth. For example, explain why autonomy and choice remain low despite policy efforts (lines 355–358).

**Response:** We have refined as per the comment given.

**Comment:** Avoid speculative statements without evidence (e.g., “medical error” as a reason for poor responsiveness, lines 372–374).

**Response:** We have refined as per the comment given.

**Comment:** Strengthen explanation for non-significant socio-demographic factors—could sample homogeneity be a reason?

**Response:** We have added sample homogeneity as one possible explanation for non-significance of socio-demographic variables.

**Comment:** Provide concrete policy implications (e.g., how training and infrastructure improvements can address low autonomy and choice scores). You could have a section for this.

**Response:** We have refined as per the comment given.

**Comment:** Expand on strengths and Limitations – The discussion should include methodological limitations, such as potential social desirability bias due to interviewer-administered surveys and facility-based sampling that may exclude home deliveries.

**Response:** We have refined as per the comment given.

**Conclusion (pp. 26–27, lines 396–410)**

**Comment:** Avoid vague statements like “relatively higher” without benchmarks.

**Response:** We have refined as per the comment given.

**Comment:** Include recommendation for integrating responsiveness indicators into routine maternal health quality assessments.

**Response:** We have the mentioned recommendation.

**Comment:** Strengthen Conclusion – While the conclusion summarizes key findings, it could better distinguish between empirical conclusions and recommendations, avoiding generalizations beyond collected data.

**Response:** We have considered reviewer’s suggestion.

**Comment:** Language: Several grammatical issues and awkward phrasing (e.g., line 55, “crucial in impact health system responsiveness”). A thorough language edit is recommended.

**Response:** We have checked for these defects and improved the manuscript.
